# Use of Edible, Medicinal, and Aromatic Plants in Various Health Disorders: A Cross-Sectional Evaluation among Inhabitants in the Area of Thrace, North-Eastern Greece

**DOI:** 10.3390/ijerph191912576

**Published:** 2022-10-01

**Authors:** Georgia-Eirini Deligiannidou, Chrysoula Kyrgou, Evangelia Nena, Vangelis G. Manolopoulos, Eugenia Bezirtzoglou, Christos A. Kontogiorgis, Theodoros C. Constantinidis

**Affiliations:** 1 Laboratory of Hygiene and Environmental Protection, Medical School, Democritus University of Thrace, 68100 Alexandroupolis, Greece; 2 Laboratory of Social Medicine, Medical School, Democritus University of Thrace, 68100 Alexandroupolis, Greece; 3 Laboratory of Pharmacology, Democritus University of Thrace-School of Medicine, 68100 Alexandroupolis, Greece

**Keywords:** nutrition, natural products, epidemiology, Mediterranean diet, aromatic and medicinal plants, Thrace, northern Greece

## Abstract

Background: Medicinal and aromatical plants (MAPs) have been historically used as traditional remedies in many cultures in Europe and globally. The aim of this study was to evaluate the use of MAPs in various health disorders in association to dietary habits and other lifestyle factors among residents in Thrace, NE Greece. Methods: Data were collected through anonymous and voluntary responses to a structured online questionnaire, via convenience (snowball) sampling. Results: The 561 responders (age: 39.7 ± 11.6 y) were mostly female (59.7%), with higher education (69.8%), working as state or private employees (55.4%), and having low/medium income (77.1%). Overall, more than 70% were using MAPs in various symptoms and common health disorders, such as chamomile against common cold and the flu. More than 20 different MAPs were being used in smaller frequencies against various conditions. Key contributing factors to the consumption of MAPs were sex (female over male), employment (employed vs. unemployed), education (higher education vs. lower) and higher Body Mass Index (overweight and obese vs. normal), while consumption of fruit, fish, and vegetables was mainly associated with the use of MAPs as common items of diet and in health disorders. Conclusions: The use of MAPs as part of the diet and as traditional remedy is present in the examined population, while particular choices seem to be affected by sociodemographic and lifestyle factors.

## 1. Introduction

The World Health Organization (WHO) European Region Action Plan 2015–2020 conveys a clear message about the importance of nutrition, and well-advised food choices in all stages of life [1]. However, the way toward tailored and comprehensive nutrition policies within the framework of public health and the implementation of resilient and efficient evaluation and monitoring systems is more than a linear path [2,3,4,5,6,7]. Dietary habits are directly related to several diseases, while malnutrition and increased weight status are included in the short list of behavioral factors responsible for more than 750,000 deaths per year [8,9]. Although effective nutrition interventions can aid the efforts for the development of disease-preventing strategies, according to the 2019 report of the European Commission, only 3% of spending is devoted to prevention across the EU (with Greece being even lower in the chart at almost 1.3%), hence narrowing the potential of effective nutrition policies [8,10]. 

In Greece, the nutritional habits have evolved alongside the political and economic scenery of the country. Although, there are several distinct dietary habits that correspond to specific parts of Greece (i.e., islandic, mainland, and mountain), the population’s characteristics overall reflect a Mediterranean diet (MD) pattern [11]. This principal link to MD in modern Greece began with the Cretan diet which captured international scientific attention as a healthy diet pattern [12,13]. In later years, alterations in the dietary habits and the use of medicinal and aromatic plants (MAPs) were subject to various external influences from Western Europe and America [14], the long recession of Greek economy [15,16,17,18] and the COVID-19 pandemic, are distressing additions to the already challenged economic, social, and ultimately dietary outlook of the country [19,20]. 

As previous research reports, a large part of the Greek population and particularly children, have moved further away from the MD in terms of fruit, vegetable, and legumes consumption frequency; however, the core characteristics of MD remained resilient and in recent years there is even more interest in the traditional and regional dietary habits [21,22,23]. Additionally, the consumption of fresh and dried herbs, including MAPs, as part of the dietary pattern in Greece, has also been identified as a beneficial contributor toward healthy and sustainable nutrition [24]. In this setting, the use of natural products has always been a significant part of the Greek diet and culture in the context of nutrition as well as therapeutics [25,26]. To this day, in many other parts of the world, MAPs are widely used as medication in various pathologies, even though a vast number of the world’s flora (estimated over 500,000 species) remains uncharted in terms of pharmaceutical potential [27]. Natural products and especially MAPs have a wide range of vitamins, minerals, antioxidants, fiber, and fatty acids that attribute to several beneficial outcomes in non-communicable diseases (NCDs) prevention [28,29,30,31]. The dietary habits of subpopulations in Greece have been the item of previous research [32,33]. However, current knowledge mostly reflects the consumption patterns of major food groups and their relation to the population’s health status. To that end, a targeted approach to the use of MAPs against symptoms and common health disorders is, to our knowledge, missing. 

Thrace is a geographical and historical region located in the North-East part of Greece. The area is characterized by cultural diversity of the population, as well as its diversity of the landscape, which changes from a marine to an alpine landscape, hence providing a vast variety of aromatic and medicinal indigenous plants [34,35,36]. To date, the number of studies documenting the traditional uses of MAPs and their products in Greece are scarce, while there is a limited number of investigations regarding the dietary habits in relation to health status in this region of Thrace in particular [37,38]. 

In this context, our work aims to describe the characteristics of MAPs consumption in the area of Thrace focusing on their use as treatment to numerous health disorders. Three main conditions were followed in this study: (i) the use of MAPs is often attributed to traditional knowledge of remedies against various pathologies, however the term “traditional use” is often linked to the elderly part of the population as well as to those located in the rural areas; (ii) advances in research over the past years have made known to the public the beneficial effects of MAPs, hence what may have started as “traditional” gained scientific focus; and (iii) MAPs constitute a part of the population’s diet pattern and are in turn affected by the overall food choices of the individuals as well as the sociodemographic and lifestyle factors. On that note, our study has the following axes of evaluation: (i) to study the use of MAPs against health disorders common among that part of the non-elderly, urban population in Thrace, (ii) to document MAPs consumption against health disorders, (iii) to identify potential sociodemographic and lifestyle factors that are related to the use of MAPs in this population sample, and (iv) to assess if the overall dietary habits of this population affect the use of MAPs. Additionally, given the fact that this study was conducted during the COVID-19 pandemic, the potential influence of this global event was also evaluated.

## 2. Materials and Methods

### 2.1. Study Design 

This work presents the data collected through an online questionnaire evaluating: (i) Sociodemographic and Lifestyle factors such as income, employment status, education level, smoking, exercise frequency, etc., of the population, (ii) use of MAPs with a focus on the type and reason for consumption, and (iii) dietary habits. The questions were provided in Greek language and assessed either via a frequency scale or by providing several answering options to the respondents. Inclusion criteria for the participation in this study included: (i) age ≥ 18 years, (ii) being a permanent resident of the region of Thrace, (iii) providing consent for data collection and analysis.

### 2.2. Questionnaire Description

In addition to general questions regarding sex, age, height, and weight, participants were asked to respond to the following questions divided in three main sections:

- Section of sociodemographic, economic, and lifestyle factors: This section included questions regarding the “residence” (rural setting: <2.000 inhabitant; semi-urban setting: 2.000–10.000 inhabitants and urban setting: >10.000 inhabitants, as defined by the Hellenic Statistical Authority (EL.STAT.) in the “Inventory of official national-level statistical definitions for rural/urban areas” by the International Labor Organization); “marital status” (single, married, in relationship/cohabitation, divorced and widowed); “education level” (primary school, gymnasium, lyceum, university, master’s degree, and doctoral); “personal income per year” (EUR < 10.000, EUR 10.000–25.000, EUR 25.000–40.000 and EUR > 40.000) (EL.STAT.); and “employment status” (unemployed, student, state employee, private employee, freelancer, part time job, farmer (as a special employment category), retired, and household). Lifestyle questions included: “smoking” (yes, no, yes occasionally, and have quit smoking for over 1, 2–5, 6–10, or 10 years); and “exercise” (never, occasionally but not frequently, frequently for less than 150 min per week, and frequently for more than 150 min per week).
- Section of use of aromatic and medicinal plants: This section included questions regarding the “frequency of consumption” of herbal beverages (never, <1 per month, 1–2 per month, 1–2 per week, 3–4 per week, >5 per week, or >2 per day); “often consumed herb” as beverage (a list of 18 common herbs was provided in addition to a free text option). The list included: chamomile (*Chamaemelum nobile*), green tea (*Camellia sinensis*), black tea (*Camellia sinensis*), mountain tea (*Sideritis* L.), mountain tea (local) (*Sideritis* L.), Tilia (*Tilia* L.), sage (*Salvia officinalis*), Melissa (*Melissa officinalis*), Aloysia (*Aloysia Juss*.), Valeriana (*Valeriana officinalis*), lavender (*Lavandula* L.), oregano (*Origanum vulgare*), rose (*Rosa* L.), fennel (*Foeniculum vulgare*), Achillea (*Achillea millefolium*), Majorana (*Origanum majorana* L.), St. John’s wort (*Hypericum perforatum*), and common dandelion (*Taraxacum officinale*); and “reason for consumption” of herbal beverages (a list of 17 conditions, symptoms, and common health disorders, leading to the consumption of herbal beverages was provided in addition to a free text option. These reasons for consumption included: Common cold, flu, digestive disorders, improving renal function, insomnia/relaxing, as stimulant, as diuretic, blood sugar lowering, lipolysis, tachycardia, muscle pain, joint pain, gallstones, improving intestinal function, dysmenorrhea, pharyngitis, and cholesterol lowering).
- Section of dietary habits assessment: This section included questions regarding the “frequency of consumption” of seven food groups (fruits, vegetables, cereals, fish, milk and products, red meat, and wine) on a frequency scale (never, 1–2 times per month, 2–3 times per month, 1–2 times per week, 3–4 times per week, 4–5 times per week, and daily); “olive oil consumption” per week (never, <2 days, at least 2 days, at least 3 days, at least 5 days, and daily); “preference of local olive oil over commercial” (yes or no); “number of meals” per day (1–2, 3, 4, 5, and >5); and “frequency of alcohol consumption” per week (never, <1 time, 1–2 times, 3–4 times, or >4 times).

### 2.3. Data Collection and Screening

The online questionnaire was disseminated via social media, and word-of-mouth communication (convenience sampling with a snowball technique) for the collection of data. Data were collected between November 2019 and mid-December 2021. It must be noted that the survey was already launched when the COVID-19 pandemic was declared by the WHO (March 2020). On that note, the study protocol as well as the questionnaire were not altered with the aim of including this particular condition, while the participants had the option of describing this or any other pathologic disorder in the free text option. A total of 568 responses were initially obtained. All responses were screened and seven were excluded from further analysis as they did not comply with the inclusion criteria. Overall, 561 responses were included in the analysis presented in the results. 

### 2.4. Statistical Analysis

All variables were appropriately coded. Based on the self-reported height and weight, BMI was calculated and classified according to the WHO criteria into the following classes: <18.5kg/m^2^ = underweight, 18.50–24.99 kg/m^2^ = normal range, 25.00–29.99 kg/m^2^ = overweight, 30.00–34.99 kg/m^2^ = obese or obese class I, 35.00–39.99 kg/m^2^ = overly obese or obese class II, and >40.00 kg/m^2^ = extreme obesity or obese class III [39]. For this study, the following steps were taken through the analysis of the use of MAPs. The initial step was the overall evaluation of positive responses regarding the use of MAPs under two conditions: (i) as a common item of the individual’s diet (also found in the manuscript as common use), and (ii) as a therapeutic choice against various symptoms and common health disorders (also found in the manuscript as therapeutic use). It must be noted that the use of other medication or therapeutic approach by the positive responders in the second condition was not an item we investigated, and that the classification of “common” and “therapeutic” use was solely used to distinct the two cases. 

Descriptive statistics (absolute frequencies and percentages) were used for ordinal or nominal variables, while mean ± SD was used for scale variables. Kruskal–Wallis (KW) test, Spearman’s correlation (SCC), Principal Component (PCA), and Odds Ratio (OR) analysis, were used for specific analyses. Descriptive statistics and KW test were used to identify differences between groups (e.g., positive responders for common use of MAPs vs. positive responders for therapeutic use of MAPs). SCC, PCA, and OR were used to identify the key contributing factors (e.g., sex, age, education) for each group. All statistical tests and outcomes were based on a significant level of 5% (α = 0.05). 

## 3. Results

### 3.1. Population Demographics 

The responders of this survey were adults (39.71 ± 11.61y, min: 18, max: 85) living mainly in urban and suburban regions of Thrace (88.1% of the total sample), mostly women (59.7%) and non-smokers (63.1%). Regarding the general socioeconomic demographics, the majority of our sample had higher education (university degree holders and higher, 69.8%), they were employed mostly as state or private employees (55.4%), had low to medium income (77.1%) (EUR < 10.000, or EUR 10.000–25.000), and were either married or in a relationship (65.5%) (Table 1). 

Although mean BMI of the participants falls within the overweight group, with no significant differences between males and females, a more precise look into groups based on BMI demonstrated that the majority of female participants belong to the normal BMI class, while the larger proportion of male participants falls into the overweight and obese BMI classes. The rates of underweight and severely obese participants are relatively low for both male and female. Approximately 20% of the participants reported regular exercise for more than 150 min per week and the majority reported moderate-to-low alcohol consumption.

### 3.2. Use of MAPs

The use of MAPs within the diet pattern as well as in the context of traditional medicine is common for many cultures [40,41,42,43,44]. As shown in Table 2, 96.4% of participants in this survey consume MAPs as an item of their diet and 72.9% reported using MAPs against one or more health disorders. In both cases, female positive responders were almost 1.5 times more than male, with similar distribution of responses across the 18–24, 25–35, and 36–45 age groups, and marital status. Additionally, positive responders belonged mainly to the classes of higher education level (university degree and higher), low/medium income (EUR < 25.000), and the unemployed participants of this survey. In all cases, the pattern of consumption (regarding the common use of MAPs and their use against health disorders) was proportionally similar with respect to the socioeconomic factors. At this step of evaluation, the participants’ classification regarding habits such as: smoking, alcohol consumption, exercise, and BMI did not reveal any essential differences with respect to the common or therapeutic use of MAPs.

The second part of this survey focused on the MAPs used by the positive responders as well as the health disorders they were used for. As shown in Table 3 chamomile (53.7%), green tea (42.6%), black tea (37.6%) and local mountain tea (29.7%) were the most commonly consumed MAPs, while in rates less than 10% of our population sample other responses included: thyme/oregano (8.0%), St. John’s Wort (7.3%), Majorana (6.9%), Valerian (6.1%), lavender (5.5%), rose (3.6%), mint (3.2%), and fennel (2.3%). It is also noted that by merging the responses of local mountain tea and mountain tea, the overall positive responses sum up to 42.3% (n = 229), ranking this choice as the third most common among the participants. Additionally, the main reasons for use, as reported by the positive responders, were common cold (47.2%), flu (17.5%) and insomnia/as a relaxant (16.0%).

**Figure 1:**
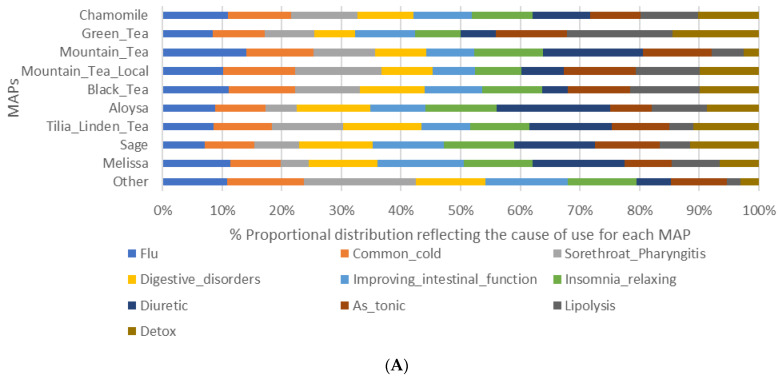

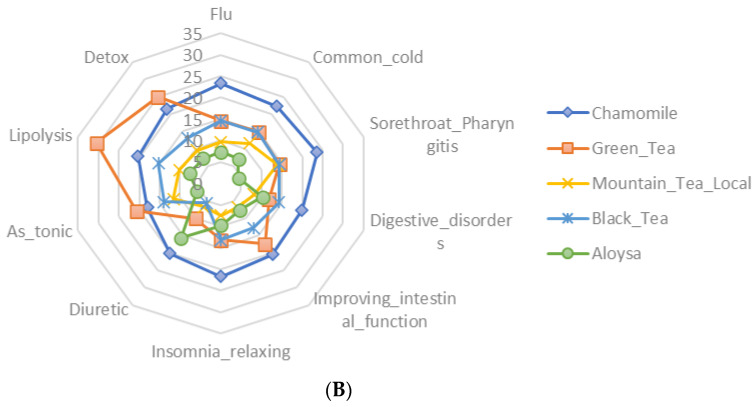
(**A**) % proportional distribution reflecting the cause of use for each MAP, (**B**) ranking of the top 5 MAPs in the conditions.

It should be mentioned that chamomile was the most common choice in seven out of ten conditions, with slightly higher rates in the cases of flu, common cold, and sore throat/pharyngitis, and a relatively equal distribution of choice among the health disorders (Figure 1A,B). However, in the cases of use “as stimulant”, “for lipolysis” and “for detoxification”, green tea ranked first in the participants’ choice, closely followed by chamomile in the cases of “detoxification” and “as stimulant” but having a bigger gap in the case of “lipolysis”. Additionally, Aloysia ranked second in the participants’ choice as a diuretic, closely following chamomile.

As mentioned previously, our study was already launching/running, when the COVID-19 pandemic began. However, we decided not to change our study protocol in order to include the condition of COVID-19 in the options of health disorders. Although the respondents had an option to describe a different pathology for which they use MAPs, we received no responses describing this condition. In this setting, we tried to determine potential variations in the overall use of MAPs before and after the pandemic declaration by the WHO [45]. 

**Figure 2:**
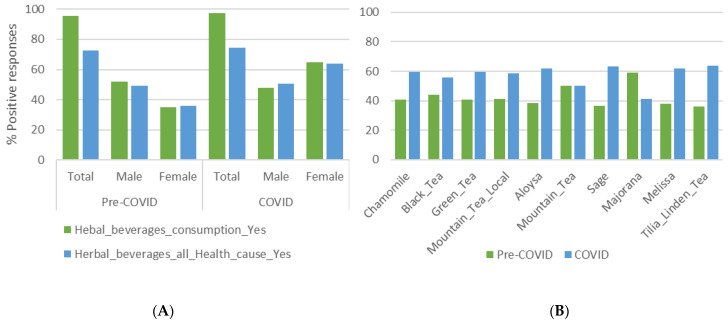
(**A**) Positive responses on the use of MAPs before and after the COVID-19 pandemic was declared, distributed by sex (male; female; total). (**B**) Positive responses on MAPs choice before and after the COVID-19 pandemic was declared.

As shown in Figure 2A, a small increase in the positive responders for the overall common use of MAPs was observed, namely from 95.34 to 97.23% as well as in various health disorders (from 72.84 to 74.53%), with the increase although present in both sexes, being more pronounced in females. This overall increase in the consumption of MAPs, was also expressed as an increase in specific MAPs commonly consumed (referred to as the choices of MAPs), which differed (*p* = 0.003) before and after the pandemic (Figure 2B).

Use of most of the MAPs was increased except for mountain tea, which remained the same (although the local variety has increased) and Majorana, which decreased. It is noteworthy that there were no changes in the overall ranking of the preferred MAPs, as previously presented (Table 2), in this before and after evaluation.

**Figure 3:**
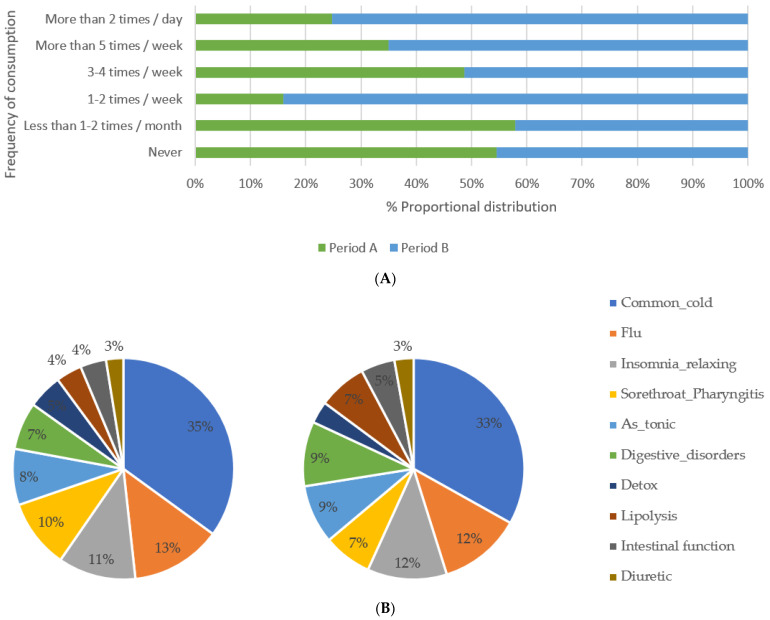
(**A**) MAPs frequency of consumption during Period A (before COVID-19) and Period B (after COVID-19) (**B**) MAPs common cause of consumption during Period A and Period B.

There was also a significant change in the frequency of consumption since the majority of positive responders reported daily and weekly consumption for the period after the pandemic was declared. For instance, over 70% of positive responders declared daily consumption in period B, while the correspondence in period A was less than 25%. Moreover, some slight changes were noted regarding the reason for consumption (Figure 3A,B). On that note, the seasonality that may have affected our results is taken into consideration as in both periods, winter and summer months are included.

In this context of evaluation, changes observed between the evaluated periods were relatively small and hence none of them was rendered statistically significant. However, it is noted that there has been a 2% increase in the positive responses regarding the use of MAPs in digestive disorders which upgraded this use from sixth to third place in the ranking, and 3% increase in the use of MAPs for lipolysis which upgraded the cause from eighth to fourth place in the ranking. Additionally, although the four major causes of use remained the same in ranking among the positive responders, a decrease 1–3% was recorded in most of them except for insomnia/relaxing.

During the next step of evaluation, factor analysis for positive responders on MAPs consumption as common use and in health disorders were analyzed separately. In both cases all variables such as region type, sex, age groups, income, education level, BMI groups, employment status, marital status, exercise, and smoking status, were included. The outcomes of the first run regarding consumption as common use revealed that five factors (out of 10) could explain the variance of our population sample up to 63.04% (Kaiser–Meyer–Olkin Measure of Sampling Adequacy (KMO) = 0.555, *p* < 0.01). Component 1 included age groups and employment status loaded very well together (loadings: 0.806 and 0.789, respectively). Component 2 included the variables of income, marital status, and education level loaded very well together (loadings: 0.759, 0.666 and 0.542, respectively), and component 3 included region type, smoking status, and BMI groups, loaded fairly well together (loadings: 0.750, 0.593 and 0.445, respectively). Notably, BMI groups were also loaded in component 1; however the loading was relatively low (0.347) compared with the other variables in this group. Gender and exercise variables had strong loadings in components 4 and 5, respectively. 

In a similar way, factor analysis for positive responders on MAPs consumption in health disorders also revealed that five factors (out of 10) could explain the variance of our population sample up to 64.72% (KMO = 0.544, *p* < 0.01). In brief, the outcomes were very similar to the initial run regarding the common use, as the first component included age groups and employment status loaded very well together with relatively higher loadings (0.826 and 0.805, respectively). Component 2 included the variables of income, marital status, and educations level, and component 3 included sex, smoking status, and BMI groups, loaded fairly well together (loadings: 0.852, 0.346, and 0.310, respectively). Notably, the smoking status was also loaded in component 4, along with region type (loadings: 0.575 and 0.814, respectively) and the BMI groups were also loaded in component 5 along with exercise (loadings: 0.595 and 0.826, respectively).

Based on the previous results, the KW test was conducted to identify statistical significance of the variables relevant to MAPs consumption either as common use or in health disorders. For this analysis the top five variables were included, namely BMI, marital status, age, smoking status, and education level. The outcomes revealed that only BMI was statistically significant (*p* = 0.020) in the case of consumption as common use, while marital status was borderline significant (*p* = 0.043) in the case of MAPs consumption for health disorders. In both cases, the Mann–Whitney test was conducted in order to identify the particular group of BMI and marital status that was relevant to the outcomes observed. Results from this analysis showed that in the case of MAPs consumption, as common use, significant differences were present in the comparison of participants in the BMI group of 18.5–24.9 kg/m^2^ against those in the BMI group of >35 kg/m^2^ (*p* = 0.030). 

Regarding the effect of marital status, marginal difference was noted in general. More specifically, in the case of MAPs consumption against health disorders, moderate differences were present in the comparison of participants in the “single” group against those in the “in relationship” group (*p* = 0.046). Additionally, SCC showed that there was a small correlation between marital status and consumption of MAPs in health disorders (*p* = 0.043). An interesting finding of SCC evaluations was that there is medium (0.303, *p* < 0.01) correlation between MAPs consumption as common use and in health disorders, which also reflects the similar patterns observed in the PCA analysis initially conducted.

As mentioned in the study design, another aim of the study was to explore the dietary habits of the participants in relation to the use of MAPs as part of their diet. On that note, the participants of this survey were asked to report on the number of meals consumed daily as well as on the frequency of consumption of eight major categories of food items, namely: fruits, vegetables, cereals (including legumes), red meat, fish (including seafood), milk (including cheese, yogurt, and dairy products), wine, and olive oil. 

**Figure 4:**
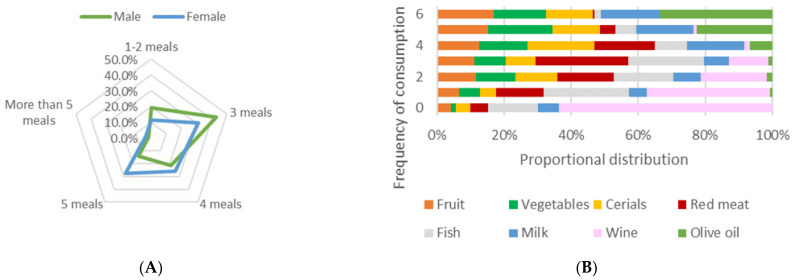
Consumed meals per day, food choices and % frequency of consumption. (**A**) Overall consumed meals per day: responses % for male and female participants, (**B**) overall food choices and % frequency of consumption for all responders (0 = never, 1 = 1–2 times per month, 2 = 2–3 times per month, 3 = 1–2 times per week, 4 = 3–4 times per week, 5 = 4–5 times per week, and 6 = daily).

As seen in Figure 4A,B, the majority of participants consume fruits, vegetables, cereals, and milk on a daily basis or at least more than 3–4 times per week on an average 3–5 meals diet. Additionally, it is more common for male participants to consume 1–3 meals per day and have more frequent consumption of red meat and cereals, whereas female participants commonly consume 4–5 meals per day and have more frequent consumption of fruit, vegetables, and olive oil. Moreover, the majority of participants responded positively on preferring the local variety of olive oil, which was observed in both male (*n* = 152, 67.3%) and female (*n* = 247, 73.7%) responders. 

The goal was to evaluate the use of MAPs as part of the participants’ diet in addition to their use in various pathologies. Hence, this initial observation of the overall eating behavior was further utilized in the study of the correlation of dietary habits with the use of MAPs. In this case, all responses were translated into a dichotomous scale reflecting consumption frequencies within a healthy diet pattern based on the WHO guidelines for healthy diet for the prevention of chronic diseases and relevant references regarding the Mediterranean diet pattern in particular [46,47,48]. In this setting, consumption within the guidelines was marked as “yes” and outside (either higher or lower) was marked as “no”. A summary of the dietary habits of the positive responders for the common use of MAPs and their use in health disorders is presented in Table 4. 

Similar analysis steps as the ones followed for the sociodemographic factors were also followed for the assessment of any associations between our sample’s dietary habits and consumption of MAPs as common use or in case of a disease or a health disorder. In brief, KW test was conducted to identify statistical significance of the dietary variables relevant to MAPs consumption. The analysis revealed that only the number of meals per day was statistically significant (*p* = 0.036) in the case of MAPs consumption in health disorders, while consumption of fruit (*p* = 0.022), vegetables (0.020), fish (0.008), and wine (*p* = 0.037) were significant in the case of common use. SCC analysis revealed the presence of positive correlations between usual MAPs consumption and consumption of fruit (0.090, *p* = 0.033) and fish (0.099, *p* = 0.019), while MAPs consumption in health disorders was correlated with the number of meals consumed per day (0.089, *p* = 0.036).

**Figure 5:**
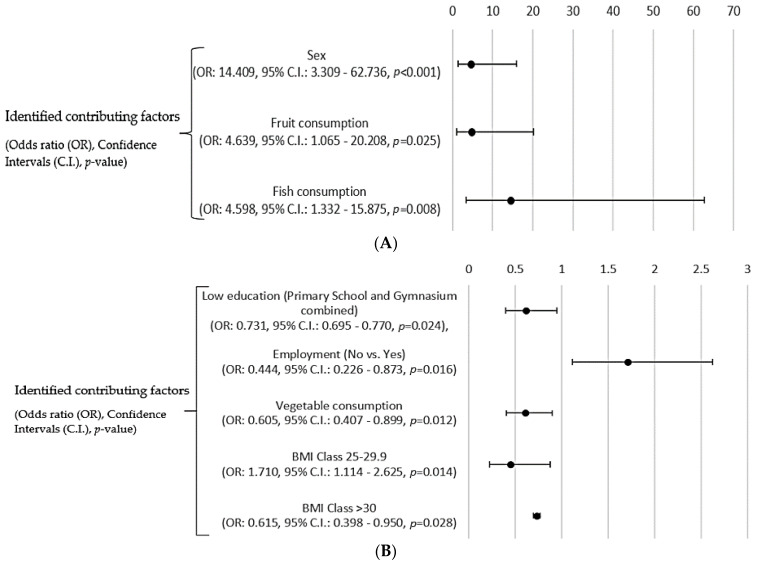
(**A**): Odds Ratio outcomes regarding the common use of MAPs, (**B**): Odds Ratio outcomes regarding the use of MAPs in symptoms and common health disorders.

As a next step, regression analysis was applied, in order to detect predictive factors for positive responses in the consumption of MAPs. Figure 5A describes the factors related to the common consumption of MAPs and Figure 5B describes the factors related to the consumption during pathologic disorders. For the common use of MAPs, sex and fruit and fish consumption were found to be predictive factors, while the use of MAPs due to different disorders is associated with the education lever (higher than basic education), employment status (unemployment), and higher BMI (overweight and obese), as well as with lower rates of vegetables consumption.

## 4. Discussion

Medicinal and aromatic plants have been an item of investigation over the past decades [24,25,26,27], and with good reason, as many of them have demonstrated beneficial characteristics for our health [25]. A number of studies have been dedicated to the support of such characteristics for several well-known plant species, some of which were also present in the results of this work. The use of chamomile is associated with the improvement of sleep quality and generalized anxiety disorders in addition to weight management and antioxidant activity [49,50]. Green tea is reported to be beneficial for the improvement of cardiometabolic risk factors such as blood pressure, glucose, and lipids [51], while the extracts of Mountain tea have been well-documented for their antioxidant and anti-inflammatory properties [52]. Furthermore, there is growing interest of the immunomodulatory activity of MAPs and a number of studies have recently provided such results [53]. In this context, plant-derived compounds such as luteolin (mountain tea, chamomile, and Aloysia-luteolin-7-O-diglucuronide) [50,52,54], apigenin (mountain tea, chamomile) [52,55], epicatechin (green and black tea) [56], naringenin (Aloysia) [57], caffeic acid (mountain tea) [58], rosmarinic acid (mountain tea) [58], and many others (Figure S1, Supplementary File) have also long emerged as individual items of investigation. These plant species have been widely distributed in various regions of Greece and thus, their role in traditional medicine and therapeutic is widely known [59,60]. 

Consequently, an essential part of research around MAPs commonly reflects the evaluation of their chemical and biological characteristics as well as their potential as agents in food production [61,62]. On that note, their role or even their presence in the population’s diet, as edible and commercial items, is rarely studied, particularly in Greece [24]. In the current study, we focused on the use of edible MAPs exploring at the same time the dietary habits of a population sample living in the area of Thrace. Our results demonstrated that the majority of our study population includes MAPs in their regular diet as over 90% of the participants reported consuming MAPs, with over 70% using MAPs as a treatment against various symptoms and health disorders. As shown by a previous study, certain socioeconomic factors impact health-related behaviors, such as high alcohol consumption (*p* = 0.030), low adherence to Mediterranean diet (*p* = 0.016), and low physical activity (*p* < 0.001) [37]. In addition to that, our study also demonstrated similar findings regarding the use of MAPs, with factors such as sex, education, employment status, food choices (namely fruit, vegetables, and fish consumption) being associated with common or therapeutic use of MAPs. Previous investigations carried in Greece and particularly in the area of Thrace have mainly focused on the diet pattern of the population in relation to their health, providing crucial insights of the social determinants implicated, but with limited information on MAPs consumption characteristics. In this context, our findings are further discussed in relation to relevant studies conducted in other countries.

An early 2004 study conducted in Norwest England including 21,923 participants reported correlations between the use of herbal supplements and sex (for females OR: 3.06, 95% C.I.:2.74–3.41) as well as lifestyle variables, such as physical activity (age and sex adjusted OR: 1.34, 95% C.I.:1.21–1.48) [63]. This finding was also observed in our population sample in the case of aromatic and medicinal plants, particularly in the case of female responders. Additionally, our findings are closely related to similar surveys conducted in recent years, also focusing on the assessment of the use of natural products in pathological conditions. In our survey we demonstrated the influence of socio-demographic factors, particularly female sex, education level, and income, which have also been reported to correspond to the use of dietary supplements. Namely, the impact of sex (females OR: 1.41, 95% C.I.: 1.05–1.90), education (Post-secondary OR: 1.60, 95% C.I.: 1.15–2.22), and income (higher income OR: 2.04, 95% CI: 1.16–3.59) have also been previously identified as predictors for the use of natural health products in studies conducted in Canada and Thailand [64,65]. 

Notably, the assessments of natural products have also been relevant during the COVID-19 pandemic as protective measures taken by the population [66,67,68,69]. The results of our study have been closely related to previous studies conducted during this period in various populations, including professional dietitians [70]. Namely, a 2021 study conducted in Turkey demonstrated that women were more common consumers of herbal medicines and within the profession of dietitians (hence participants of higher education) 46.1% used herbal medicine during the COVID-19 pandemic [70]. In relation to these findings, our study also showed an increased use of MAPs on the period immediately after the declaration of the COVID-19 pandemic, which was also more pronounced with the female participants. Additionally, our results demonstrated a relatively small increase in the consumption of MAPs during the period after the COVID-19 declaration which is indicative of the fact that dietary habits need time to change and are also resilient once they become routine. 

A relatively divergent finding related to our outcomes is the effect of education level. A study conducted in 2014 in Kenya showed that lower education was associated with the use of herbal medicine by women during pregnancy [71], as opposed to our findings that associated higher education. These variations can be explained by the specific characteristics of that subpopulation (pregnant women), as well as by the overall differences in dietary and lifestyle factors between an African and a European population. However, also related to our findings and despite the aforementioned differences, this study also demonstrated the relevance of herbal medicine use in digestive disorders and respiratory tract infections, which were among the most common causes of use in our study as well [71]. Additionally, a prospective cohort study conducted in China, before the pandemic, also found that mothers with lower family income were more likely to consume Chinese herbal medicines postpartum [72]. This can also be related to our findings that unemployment is related to the use of aromatic and medicinal plants. It is worth mentioning that, although there are several cultural, social, and economic differences between the Chinese population and our study’s responders, in both cases there is a rich record of traditional use in herbal remedies, which seems to be passing to the next generations. In a similar way, our results are also in line with previous studies demonstrating higher consumption of fruit and vegetable in the positive responders of herbal supplements [73]. Moreover, a previous study in 2019 focusing on the Thai worker population showed that obesity was not identified as a predictor for the use of traditional and herbal medicine [74]. This report is also associated with our findings regarding the association of normal BMI in the use of MAPs in health disorders.

It should be noted that our study has certain limitations. The method of data collection has the downside of limited representability of the population of Thrace in our sample, such as elder population and people without access to online services. On that note, it must be highlighted that the outcomes of this study cannot be generalized as typical trades of the overall population in Thrace and can only reflect this particular part of the population, although several characteristics may remain similar. Additionally, given the circumstances of the time of the study and the voluntary nature of participation, our ability to expand the recruitment of participants and to make the responders interested in the study’s question and therefore reply to the invitation to participate was difficult and narrow.

Thus, this study should be viewed as an attempt, the first of its kind of this scale in this region and in general in Greece, to assemble data connecting the use of aromatic and medicinal plants in health disorders. Here we also briefly discussed the relevance of our findings with previous research conducted in other parts of the world. This might also be a key remark, reflecting a more global relation of aromatic and medicinal plants use to the population rather than the implemented diet pattern such as Mediterranean diet in the case of Greece.

## 5. Conclusions

In conclusion, the use of MAPs as part of the usual diet and as a traditional remedy is common practice in the population of Thrace, NE Greece, while choices are significantly affected by sociodemographic and lifestyle factors. An essential step forward would be the social awareness regarding the use of MAPs in pathological conditions, under the framework of benefits as well as safety, particularly focusing on age groups for which literature is scarce in this context, such as children. Additionally, given the high rates of positive responses in our study as well as previous studies, particularly in the cases of pathologies, the necessity of disclosure of the use of such products to health care providers is a subject that research efforts should further discuss.

## Figures and Tables

**Table 1:** General demographics and socioeconomic distribution of the population.

		Male: 226 (40.3%)	Female: 335 (59.7%)	Total: 561 (100%)
Age	Mean ± SD	40.42 ± 11.26	39.23 ± 11.83	39.71 ± 11.61
Age groups	18–24	17 (7.6%)	35 (10.6%)	52 (9.4%)
	25–35	57 (25.6%)	103 (31.3%)	160 (29.0%)
	36–45	76 (34.1%)	87 (26.4%)	163 (29.5%)
	46–55	49 (22.0%)	79 (24.0%)	128 (23.2%)
	56–65	21 (9.4%)	22 (6.7%)	43 (7.8%)
	>65	3 (1.3%)	3 (0.9%)	6 (1.1%)
Marital Status	Married	114 (50.4%)	173 (51.6%)	287 (51.2%)
	Single	73 (32.3%)	83 (24.8%)	156 (27.8%)
	In a relationship/cohabitation	23 (10.2%)	57 (17.0%)	80 (14.3%)
	Divorced	16 (7.1%)	15 (4.5%)	31 (5.5%)
	In widowhood	0 (0.0%)	7 (2.1%)	7 (1.2%)
Education	Primary/lower secondary school	9 (3.9%)	5 (1.5%)	14 (2.5%)
	Upper secondary school	66 (29.2%)	90 (26.9%)	156 (27.8%)
	University graduate	90 (39.8%)	122 (36.4%)	212 (37.7%)
	Master’s degree	49 (21.7%)	91 (27.2%)	140 (25.0%)
	PhD degree	12 (5.3%)	27 (8.1%)	39 (7.0%)
Employment	Unemployed	8 (3.5%)	35 (10.5%)	43 (7.7%)
	Student	18 (8.0%)	37 (11.1%)	55 (9.8%)
	Private employee	40 (17.7%)	39 (11.7%)	79 (14.1%)
	State employee	106 (46.9%)	125 (37.4%)	231 (41.3%)
	Part time job	6 (2.7%)	22 (6.6%)	28 (5.0%)
	Freelancer	34 (15.0%)	46 (13.8%)	80 (14.3%)
	Farmer/Breeder	6 (2.7%)	9 (2.7%)	15 (2.7%)
	Retired/Household	8 (3.5%)	21 (6.3%)	29 (5.2%)
Family Income	<10,000	42 (18.9%)	91 (28.4%)	133 (24.5%)
	10,000–25,000	122 (55.0%)	163 (50.9%)	285 (52.6%)
	25,000–40,000	52 (23.4%)	59 (18.4%)	111 (20.5%)
	>40,000	6 (2.7%)	7 (2.2%)	13 (2.4%)
Smoking Status	No	106 (46.9%)	192 (57.3%)	298 (53.1%)
	Yes	76 (33.6%)	87 (26.0%)	163 (29.1%)
	Yes, occasionally	19 (8.4%)	25 (7.5%)	44 (7.8%)
	Quit for 1–5 years	5 (2.2%)	13 (3.9%)	18 (3.2%)
	Quit for >6 years	20 (8.8%)	18 (5.4%)	38 (6.8%)
Alcohol Consumption	Never	45 (19.9%)	81 (24.2%)	126 (22.5%)
	<1 time/week	96 (42.5%)	165 (49.3%)	261 (46.5%)
	1–2 times/week	54 (23.9%)	73 (21.8%)	127 (22.6%)
	3–4 times/week	25 (11.1%)	11 (3.3%)	36 (6.4%)
	> 4 times/week	6 (2.7%)	5 (1.5%)	11 (2.0%)
Exercise	Never	33 (14.6%)	57 (17.0%)	90 (16.0%)
	Occasionally but not often	96 (42.5%)	136 (40.6%)	232 (41.4%)
	Regularly, <150 min/week	51 (22.6%)	78 (23.3%)	129 (23.0%)
	Regularly, >150 min/week	46 (20.4%)	64 (19.1%)	110 (19.6%)
BMI	Mean ± SD	27.41 ± 4.04	25.97 ± 5.61	26.55 ± 5.09
BMI Groups	<18.5	0 (0.0%)	6 (1.8%)	6 (1.1%)
	18.5–24.9	75 (33.8%)	164 (49.8%)	239 (43.4%)
	25–29.9	91 (41.0%)	90 (27.4%)	181 (32.8%)
	30–34.9	50 (22.5%)	48 (14.6%)	98 (17.8%)
	>35	6 (2.7%)	21 (6.4%)	27 (4.9%)

**Table 2:** Population demographics of positive responders.

		Common Use of MAPs, N (%)	Therapeutic Use of MAPs, N (%)
Sex	Male	208 (38.4%)	162 (39.6%)
	Female	333 (61.6%)	247 (60.4%)
Age groups	18–24	49 (9.2%)	40 (10.0%)
	25–35	156 (29.3%)	115 (28.7%)
	36–45	154 (28.9%)	120 (29.9%)
	46–55	125 (23.5%)	90 (22.4%)
	56–65	42 (7.9%)	30 (7.5%)
	>65	6 (1.1%)	6 (1.5%)
Single ^(1)^	No	278 (65.3%)	215 (64.8%)
	Yes	148 (34.7%)	117 (35.2%)
High education^(2)^	No	160 (29.6%)	129 (31.5%)
	Yes	381 (70.4%)	280 (68.5%)
Employment ^(3)^	No	72 (14.8%)	61 (16.8%)
	Yes	416 (85.2%)	303 (83.2%)
Medium to high income and above ^(4)^	No	402 (77.0%)	302 (76.8%)
	Yes	120 (23.0%)	91 (23.2%)

^(1)^: The groups of “Married” and “In relationship/cohabitation” were merged against all other groups; ^(2)^: the groups of “University graduate” and higher were merged against all other groups; ^(3)^: the groups of “Unemployed” and “Retired/Household” were merged against all other groups, and the group of “Students” was excluded; ^(4)^: the income groups were divided into two smaller subgroups: less than EUR 25,000 and more than EUR 25,000/annually.

**Table 3:** Distribution of main MAPs used and causes of use.

Main MAPs Used	Positive Responses N (%) *	Main Causes of Use	Positive Responses N (%) *
Chamomile	301 (53.7)	Common cold	265 (47.2)
Green tea	239 (42.6)	Flu	98 (17.5)
Black tea	211 (37.6)	Insomnia/as a relaxant	90 (16.0)
Mountain tea (local)	167 (29.8)	Digestive disorders	66 (11.8)
Aloysia	102 (18.2)	As a stimulant	66 (11.8)
Common sage	96 (17.1)	Sore throat/pharyngitis	65 (11.6)
Tilia (linden tea)	75 (13.4)	For lipolysis	45 (8.0)
Melissa	71 (12.7)	Intestinal function	35 (6.2)
Other	71 (12.7)	For detoxification	31 (5.5)
Mountain tea	62 (11.1)	As a diuretic	21 (3.7)

* Percentages are calculated based on the overall sample of 561 participants.

**Table 4:** Dietary habits of positive responders in the use of MAPs.

		Common Use of MAPs		Therapeutic Use of MAPs	
		Male N (%)	Female N (%)	Male N (%)	Female N (%)
Fruit	No	147 (70.7%)	210 (63.1%)	121 (74.7%)	157 (63.6%)
	Yes	61 (29.3%)	123 (36.9%)	41 (25.3%)	90 (36.4%)
Vegetables	No	157 (75.5%)	213 (64.4%)	127 (78.4%)	163 (66.5%)
	Yes	51 (24.5%)	118 (35.6%)	35 (21.6%)	82 (33.5%)
Cereals	No	133 (64.3%)	200 (60.2%)	99 (61.5%)	150 (61.0%)
	Yes	74 (35.7%)	132 (39.8%)	62 (38.5%)	96 (39.0%)
Red meat	No	23 (11.1%)	20 (6.0%)	16 (9.9%)	12 (4.9%)
	Yes	185 (88.9%)	312 (94.0%)	146 (90.1%)	234 (95.1%)
Fish	No	113 (54.3%)	184 (55.8%)	87 (53.7%)	138 (56.3%)
	Yes	95 (45.7%)	146 (44.2%)	75 (46.3%)	107 (43.7%)
Milk	No	107 (51.4%)	164 (49.4%)	88 (54.3%)	118 (48.0%)
	Yes	101 (48.6%)	168 (50.6%)	74 (45.7%)	128 (52.0%)
Wine	No	4 (1.9%)	2 (0.6%)	2 (1.2%)	2 (0.8%)
	Yes	203 (98.1%)	328 (99.4%)	159 (98.8%)	242 (99.2%)
Olive oil	No	145 (69.7%)	232 (69.7%)	117 (72.2%)	170 (68.8%)
	Yes	63 (30.3%)	101 (30.3%)	45 (27.8%)	77 (31.2%)

## Data Availability

All relevant data supporting this research which are not presented either in the manuscript or the Supplementary Section are in the possession of the corresponding author.

## Supplementary Materials

The following supporting information can be downloaded at: https://www.mdpi.com/article/10.3390/ijerph191912576/s1, Figure S1: Structure of the major phytoconstituents found in the commonly consumed MAPs.
