# Supplementary material for: Use of Edible, Medicinal, and Aromatic Plants in Various Health Disorders: A Cross-Sectional Evaluation among Inhabitants in the Area of Thrace, North-Eastern Greece
Source: Int J Environ Res Public Health. 2022 Oct 1;19(19):12576. doi: 10.3390/ijerph191912576 (PMC9566107; doi:10.3390/ijerph191912576)
Supplement: Supplementary file 1 [file ijerph-19-12576-s001.zip › ijerph-1947324-supplementary.pdf]

Figure S1: Structure of the major phytoconstituents found in the commonly consumed MAPs.

**MAPs common name (*scientific name*)**	**Names of the major phytoconstituents**	**Structure of the major phytoconstituents**
Chamomile (*Chamaemelum nobile*)	Rosmarinic acid	
	Caffeic acid	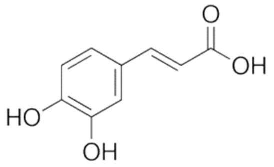
	Apigenin	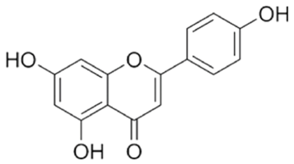
	Luteolin	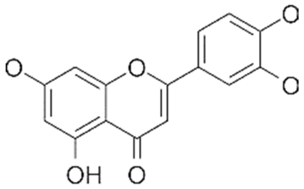
Green Tea and Black Tea (*Camellia sinensis*)	Catechin	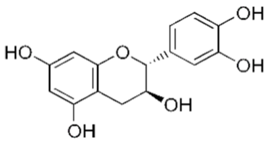
	Epigallocatechin Gallate	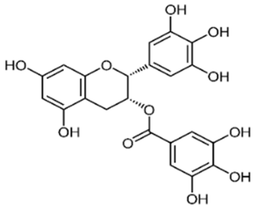
	Theaflavins	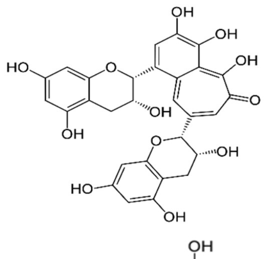
	Epicatechin Gallate	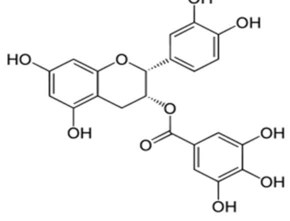

Mountain Tea (*Sideritis L.)*	Apigenin	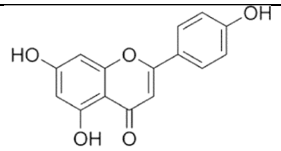
	Luteolin	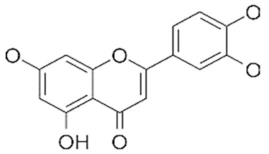
	5-Caffeoylquinic acid	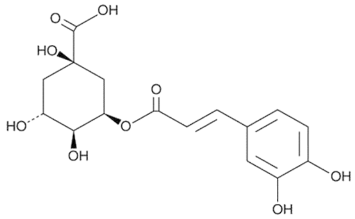
	p-Coumaric acid	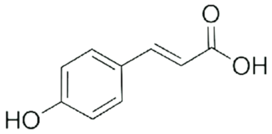
Aloysa (*Aloysia citrodora*)	Apigenin	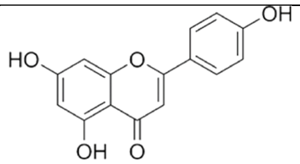
	Catechin Gallate	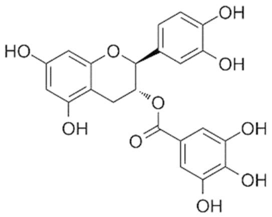
	Limonene	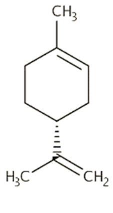
	Citronellol	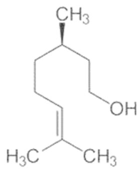
